# Deep recurrent models for forecasting infectious diseases

**DOI:** 10.3389/fpubh.2025.1726819

**Published:** 2025-12-18

**Authors:** Mai Alzamel

**Affiliations:** Department of Computer Science, College of Computer and Information Sciences, King Saud University, Riyadh, Saudi Arabia

**Keywords:** deep learning, forecasting, Google Trends, infectious diseases, recurrent neural networks

## Abstract

**Introduction:**

Infectious diseases present significant challenges to global healthcare systems due to their rapid spread and associated profound health implications. Early detection of unusual increases in case numbers is crucial for achieving efficient resource allocation and effective response planning.

**Method:**

Therefore, this research proposes and develops a time series predictive framework based on long short-term memory (LSTM), bidirectional LSTM (BiLSTM), and gated recurrent unit (GRU) neural network models to forecast the number of COVID-19 cases in Saudi Arabia and detect any unusual increase in cases. Google Trends and time series data for search terms, including “fever,” “COVID,” and “cough,” serve as input, enabling models to detect the temporal patterns associated with a surge in cases. The framework is specifically designed to model temporal dependencies in sequential data, allowing the identification of early signs of anomalies in COVID-19 case trends. Therefore, we propose training the models on preprocessed time series data while adjusting for time lags to improve predictive accuracy. Evaluations of performance are conducted using mean square error (MSE) and F1-score metrics.

**Results and discussion:**

The experimental results demonstrate that BiLSTM returns the highest F1-score of 0.83 for the term “COVID”, while LSTM and GRU reach 0.73 and 0.77, respectively. Moreover, BiLSTM outperforms LSTM and GRU at all early time lags for the search terms “fever” and “cough”. The results reveal the F1-scores for the term “fever” to be 0.77, 0.62, and 0.5 for BiLSTM, GRU, and LSTM, respectively. Whereas, the F1-scores for the search term “cough” are 0.62, 0.62, and 0.5 for BiLSTM, GRU, and LSTM, respectively. Although BiLSTM incurs higher computational costs, LSTM and GRU offer efficient alternatives to deliver rapid execution. These results highlight the effectiveness of deep learning models in instances of early anomaly detection, supporting timely healthcare interventions and advancing the development of real-time monitoring systems.

## 1 Introduction

Contemporary expansion in the availability of digital data is affording new opportunities for public health surveillance. Online search query data represents an exciting source that can reflect public interest and concern regarding symptoms associated with infectious diseases, including cases of both influenza and COVID-19 (1, 2). Contagious respiratory illnesses, such as COVID-19, continue to be among the most significant public health issues attracting high-priority care because of their high level of transmissibility. Frequently, unexpected outbreak events can complicate healthcare resource planning, due to the associated sudden surges in patient numbers, particularly in regions where healthcare infrastructure may already be less than robust (3). Recent research has increasingly leveraged Google Trends data to forecast COVID-19 and influenza-like diseases (ILIs) cases across different countries. The majority of these studies have been conducted in Western countries, specifically Europe (4–10) and the United States (11–16), with fewer being undertaken in regions such as the Middle East (17, 18), India (19, 20), China (21, 22), and Australia (23). Thus, studies in Saudi Arabia remain limited, resulting in a need to adopt and verify the available models in this context. This requires the completion of more localized studies designed to integrate search trends with other real-world data to improve early outbreak detection.

In the literature, Su et al. (24) proposed a self-adaptive AI model (SAAIM) that combines eXtreme Gradient Boosting (XGBoost) and Seasonal Autoregressive Integrated Moving Average (SARIMA) models to predict influenza cases in Chongqing, China. SAAIM outperformed conventional models, with mean absolute percentage errors (MAPEs) of 11.9, 7.5, and 11.9% for the years 2014–2016, 2017, and 2018, respectively. Prasanth et al. (3) proposed a hybrid gray wolf optimizer (GWO)-LSTM model to estimate COVID-19 cases in India, the United States, and the United Kingdom. Based on Google Trends and the European Center for Disease Prevention and Control (ECDC) data, the model significantly reduced the MAPE by 98% compared with ARIMA. Murayama et al. (25) proposed a multi-task learning model for influenza forecasting across five countries: the United States, Japan, Australia, England, and France, employing a combination of historical ILI data and Google Trends. The model used GRUs with a search query-aware attention mechanism to capture search queries in a latent way. The multi-task model performed significantly better than baseline models, with R^2^ values of approximately 0.8 to 0.9 for most one-week-ahead forecasts and also for multi-week forecasts. Lampos et al. (7) investigated the use of online search query frequencies to track the prevalence of COVID-19 across several nations, proving that such data can forecast confirmed cases and deaths approximately 16.7 and 22.1 days ahead of official reports. They highlighted the relevance of rarer symptoms as predictors by introducing unsupervised modeling approaches grounded in symptom classifications from health organizations. Transfer learning, where models were trained on data from Italy (an advanced epidemic region) and then modified for other countries, was a fundamental component of their approach. The study improved predicted accuracy, highlighting the potential for online search behavior to serve as an early indicator in the context of public health surveillance by utilizing current data from areas experiencing more advanced epidemics. Morris et al. (10) presented a methodological approach aimed at improving the accuracy of US ILI rate forecasting. They used neural network (NN) architectures to estimate ILI rates, merging web search activity time series and historical ILI data. These models proved significant in incorporating Bayesian layers, delivering forecasts with uncertainty intervals, thereby enhancing predictive confidence. The study reported that the best-performing model, termed the iterative recurrent neural network (IRNN), achieved a 10.3% reduction in MAE and a 17.1% improvement in skill scores for both nowcasting and forecasting tasks across four consecutive flu seasons.

Boulesnane et al. (26) developed a new classification model for ILI detection based on sentiment analysis and one-dimensional CNN (1D-CNNs) from Algerian Arabic Facebook posts. It utilized the following tokenization methods, including term frequency-inverse document frequency (TF-IDF) and bag-of-words (BoWs), to extract significant features. Evaluations were conducted using a 5-fold cross-validation method, with metrics such as the F1-score, which delivered a peak score of 96.6%. Elad et al. (27) examined Bing searches for COVID-19 symptoms from English users and determined that, early in the pandemic, searches for “fever” and “cough” correlated most significantly with later COVID-19 case numbers. These results suggest the potential for developing COVID-19 forecasting models that utilize symptom-related search activity as a predictive indicator. Moreover, the study proposed a technique for identifying local outbreaks, based on previous work (28, 29), that requires minimal training data. Initially, obtaining an area under the curve (AUC) of approximately 0.82, the anomaly detection system provided the UK healthcare system with an estimated one-week lead time in anticipating case increases. However, in the second phase of the epidemic, performance dropped to roughly 0.70, later becoming non-significant.

Consequently, this research aims to leverage deep learning techniques to develop predictive models that utilize Google Trends data to forecast COVID-19 cases and identify anomalies in the number of cases in Saudi Arabia, with a specific focus on the Riyadh region. Saudi Arabia has 13 Regions, of which the Riyadh region has the largest population (30). We adapt the method proposed by (7), mapping deep learning models with data based on weekly searches in the Riyadh region, using Google Trends search data. We also combined the adapted method with confirmed COVID-19 cases from historical records. These models aim to educate healthcare professionals in the early stages of a health crisis, providing them with sufficient time to prepare and resolve any shortages in staff or equipment. Thus, this research provides several core contributions to the field of disease outbreak prediction by using online search data and deep learning models: Firstly, it combines deep learning models—specifically LSTM (31), BiLSTM (32), and GRU (33)—with Google Trends search data to predict COVID-19 outbreaks in the Riyadh region in real-time. Secondly, the study investigates the use of LSTM, BiLSTM, and GRU models with a one-week temporal lag update, while identifying any lags between public interest and real case reporting. Thirdly, the study implements an effective anomaly detection mechanism based on statistical thresholds derived from mean and standard deviations in predicted cases. Finally, the work comprehensively compares the LSTM, BiLSTM, and GRU models by evaluating their performance using metrics such as the MSE and F1-scores. The main contribution of the study is as follow.

1. Deep Learning–Based Prediction: We integrate deep learning models—LSTM, BiLSTM, and GRU—with Google Trends search data and historical COVID-19 case records to predict outbreak dynamics in the Riyadh region in real time.
2. Temporal Lag Analysis: We investigate the relationship between public search interest and reported COVID-19 cases by incorporating a one-week temporal lag, providing insights into early outbreak signals.
3. Anomaly Detection Mechanism: We implement a statistical anomaly detection approach using mean- and standard deviation–based thresholds on predicted cases, enabling the identification of unusual outbreak patterns.

## 2 Materials and methods

**Figure 1:**
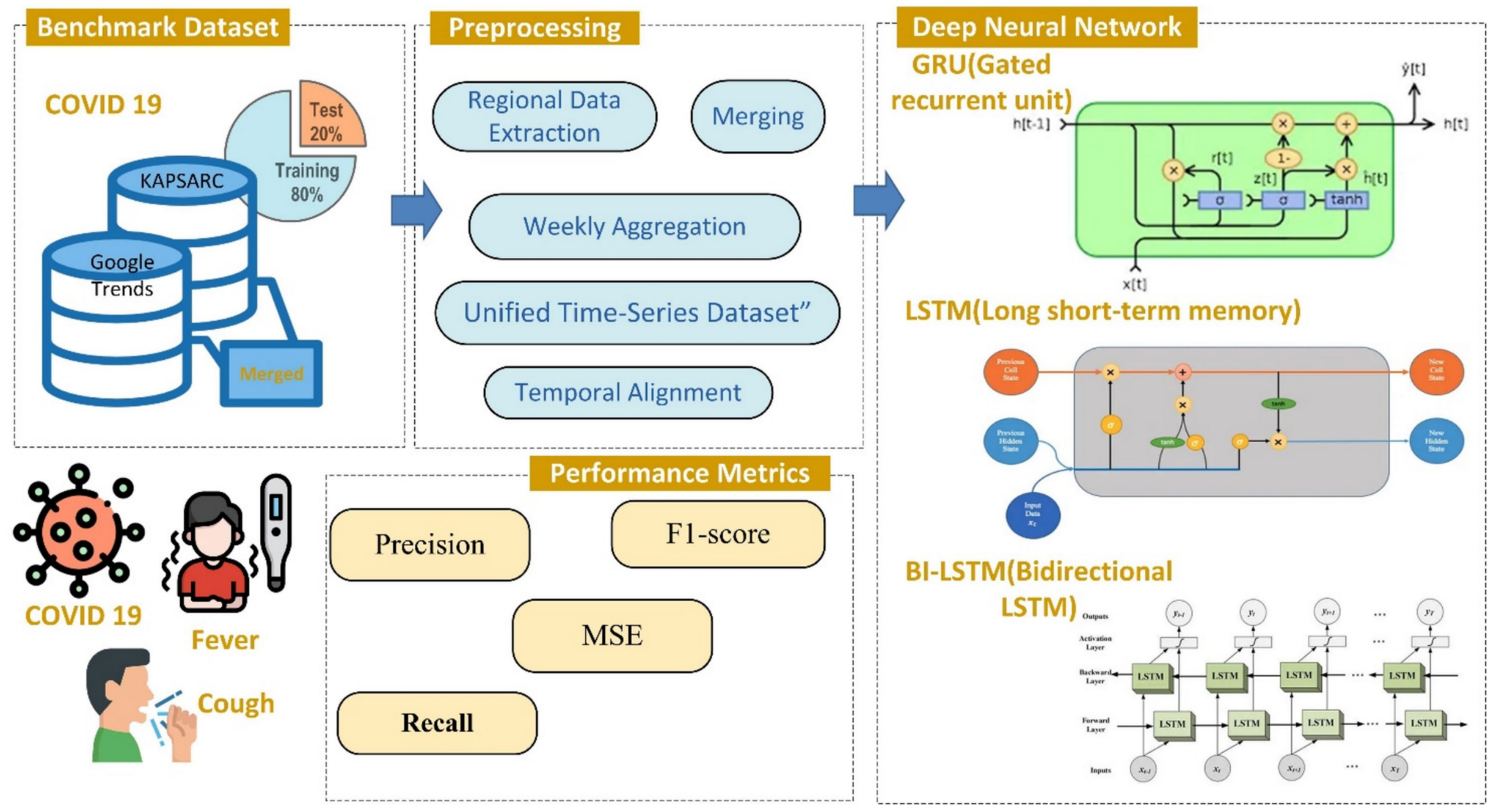
Workflow for the proposed methodology.

This section outlines the proposed methodology for predicting COVID-19 cases and detecting anomalies in the number of COVID-19 cases in Riyadh, Saudi Arabia. The methodology includes data collection, data preprocessing, model architecture and training, and model testing and evaluation stages. These stages interact to guarantee the robustness and accuracy of the developed models. The workflows in each of these sequential stages are visually represented in Figure 1.

### 2.1 Data collection

In deep learning, choosing an appropriate training dataset is essential for developing an intelligent prediction model. The choice of benchmark dataset has a significant effect on the performance of a computational model. In this study, we utilize two highly reliable datasets to validate our computational model, namely the Google Trends API (34) and KAPSARC (35). The search terms are carefully defined to capture a wide range of symptoms and concerns for COVID-19, including “fever,” “cough,” and “COVID.” Google Trends provides real-time data on general public interest and behavior, collected through search queries, whereas KAPSARC offers trusted epidemiological information on COVID-19 cases in Riyadh, Saudi Arabia. These datasets contain fundamental characteristics, including key dates, places, and the number of daily cases. To increase the temporal homogeneity of these datasets, both data sources are normalized to weekly times to match the predictive requirements. The fusion of these two datasets provides numerous insights, integrating epidemiological information with behavioral data, which can be used to build reliable predictive models.

For the training dataset, Google Trends data was collected for the period from January 3, 2021, to December 31, 2021. This time period was chosen as it overlaps with a significant increase in internet searches about COVID-19, and corresponds to the rise in official case reports in the area. The data includes weekly search interest levels from all 13 regions within Saudi Arabia, providing a comprehensive overview of search behaviors throughout the country. For model evaluation, we utilized a testing dataset comprising Google Trends data from February 28, 2022, to October 9, 2022. This dataset enables us to assess the models’ predictive capabilities on historical data before the training period, thereby evaluating their generalized performance. The process of collecting data in every region separately is a huge logistical task. Each dataset at a regional scale needs to be downloaded and extracted independently, then combined into a single unified dataset that contains all regions. This process is time-consuming and demands careful attention to ensure data integrity. Additionally, while the Google Trends data is organized every week, the official COVID-19 case data for Riyadh is provided by KAPSARC in daily counts. To ensure temporal alignment between the generated datasets, we aggregated the daily number of cases into weekly totals, thereby synchronizing the datasets for consistent modeling.

### 2.2 Data preprocessing

Robust data preprocessing is essential for transforming raw inputs into a consistent format suitable for deep learning. The workflow begins with cleaning and restructuring the KAPSARC dataset by splitting semicolon-separated fields, assigning clear column names, and filtering records to retain only daily case entries. The “Date” field is converted to datetime format and used as the DataFrame index, while the “Cases (person)” values are cast into numeric format with appropriate error handling. To maintain temporal consistency with Google Trends, the daily COVID-19 cases are aggregated into weekly totals.

The Google Trends data undergoes parallel preprocessing. Missing values in both datasets are assessed using weekly completeness checks. Weeks with missing search values are imputed via linear interpolation, and missing epidemiological values are forward-filled due to their cumulative reporting nature. All features (13 regions × 3 keywords) are then scaled using Min-Max normalization to ensure equal contribution across regions. Because Google Trends is subject to sampling variability, each regional weekly series is downloaded three times and averaged to reduce noise.

Finally, the preprocessed KAPSARC and Google Trends datasets are aligned, merged, and stripped of redundant columns to form a unified weekly dataset containing the week index, weekly COVID-19 cases in Riyadh, and 39 Google Trends features. This standardized, noise-reduced dataset serves as the final input for modeling and forecasting.

### 2.3 Correlation analysis across the 13 regions

To justify the inclusion of Google Trends data from all 13 regions during the data collection phase, we conducted a Pearson correlation analysis between weekly COVID-19 confirmed case counts and the Google Trends search terms (“COVID,” “fever,” and “cough”) for each region. This analysis was performed using the fully preprocessed and temporally aligned weekly datasets. Table 1 presents the correlation coefficients for each region.

**Table 1:** Pearson correlation between search terms and weekly COVID-19 cases across 13 regions.

Region	COVID search term	Fever search term	Cough search term
Riyadh	0.86	0.79	0.74
Makkah	0.71	0.63	0.58
Eastern Province	0.69	0.61	0.56
Medina	0.66	0.59	0.53
Qassim	0.55	0.48	0.44
Asir	0.51	0.47	0.4
Tabuk	0.49	0.43	0.39
Hail	0.46	0.41	0.35
Jazan	0.44	0.39	0.33
Najran	0.39	0.34	0.3
Al-Baha	0.37	0.33	0.28
Northern Borders	0.35	0.29	0.26
Al-Jouf	0.33	0.28	0.23

The results show that Riyadh exhibits the strongest correlation values across all search terms, indicating that search behavior in Riyadh most closely tracks the real epidemiological trend of COVID-19 cases. In contrast, regions with lower population density, such as Al-Baha, Najran, and the Northern Borders, display weaker correlations and more noise. Including these regions within the predictive model did not improve performance and tended to dilute the predictive signal. Based on this correlation evidence, we selected Riyadh as the primary region for the forecasting models, as it provides the most reliable and representative relationship between public search activity and actual case trends.

### 2.4 Proposed model architecture and training

In this paper, we implement and train three complex Recurrent Neural Networks (RNNs), namely LSTM (31), BiLSTM (32), and Gated Recurrent Unit GRU (33). The proposed approach is to construct separate models for each specific search term, including “fever,” “cough,” and “COVID.” Each of the models traces the unique temporal dependencies associated with a particular search query. At the end of model training, forecasted values are shifted based on a time lag of 1 to 4 weeks. This adjustment is crucial for determining the optimal time shift and maximizing model performance. Through the process of refining predictions and evaluating their effectiveness, we ensure that models are highly adapted and capable of accurately representing true data patterns, thereby enhancing forecast accuracy and efficiency. The following subsections explain this proposed methodology in depth.

#### 2.4.1 Training models

First, the LSTM model is particularly suited for time series data with long-distance dependencies. In this study, a many-to-one LSTM architecture is used to predict the weekly number of COVID-19 cases. For each time point, the input vector comprises 14 entries: 13 entries represent Google Trends search data across the 13 regions of Saudi Arabia, and the remaining entry represents the actual number of cases for the current week. LSTM learns these inputs through its internal gates, namely forget, input, and output gates, allowing the preservation and update of relevant information through time. The following equations govern the internal computations of the LSTM cell at the time step.


$$f_{t}=\sigma (W_{f}.[h_{t-1},x_{t}]+b_{f})$$ (1)

$$i_{t}=\sigma (W_{i}.[h_{t-1},x_{t}]+b_{i})$$ (2)

$${\tilde{∁}}_{t}=\tanh(W_{C}.[h_{t-1},x_{t}]+b_{C})$$ (3)

$$C_{t}=f_{t}*C_{t-1}+i_{t}*{\tilde{∁}}_{t}$$ (4)

$$o_{t}=\sigma (W_{o}.[h_{t-1},x_{t}]+b_{o})$$ (5)

$$h_{t}=o_{t}*\tanh(C_{t})$$ (6)

Where $x_{t}$ is the input vector at time t, $h_{t-1}$ represents the previous hidden state, $C_{t}$ is the current cell state, *σ* denotes the sigmoid activation functions, and tanh refers to the hyperbolic tangent activation functions. The weight matrices W and bias vectors b are parameters learned during training. The Equations 1–6 enable the LSTM to selectively update, retain, or forget information at each step, making it ideal for capturing temporal dependencies in sequential data. In our implementation, three separate LSTM models are constructed, each dedicated to one of the primary COVID-19-related search terms: fever, cough, and COVID-19. The input matrix *x* and the corresponding output vector *y* span *t* time points, specifically weeks. The LSTM model is trained over several epochs, and for each epoch, the MSE between the predicted and actual values of cases is calculated and minimized. Through iterative training of the model, the weights can be refined, yielding accurate predictive results, while temporal data can be processed accurately and efficiently.

Secondly, the BiLSTM model enhances the standard LSTM’s ability by incorporating bidirectional processing, which allows for the inclusion of both past and future contexts within the data sequence. This bidirectional model enhances the model’s ability to learn complex temporal patterns. For the three search terms—“fever,” “cough,” and “COVID,” a dedicated BiLSTM model is constructed. The input to the BiLSTM is time series data of the selected search terms from areas with the highest correlation with Riyadh, represented by an array denoted by x, with a focus on relevance and predictive performance. The BiLSTM architecture consists of two LSTM layers, which take the latent data contents in both forward and backward directions, as shown in Equations 7, 8. Outputs from the two directions are combined, resulting in a comprehensive representation that leverages information from both temporal directions, as shown in Equation 9.


$$\vec{h_{t}}=\mathrm{LST}M_{\text{forward}}(x_{t})$$ (7)

$$\overset{\leftarrow }{h_{t}}=\mathrm{LST}M_{\text{backward}}(x_{t})$$ (8)

$$h_{t}=[\vec{h_{t}};\overset{\leftarrow }{h_{t}}]$$ (9)

Where $\vec{h_{t}}$ is a forward LSTM, $\overset{\leftarrow }{h_{t}}$ is a backward LSTM, and ; denotes vector concatenation. This bidirectional representation allows the model to utilize both past and future information in sequence learning. In a manner analogous to the LSTM model, the BiLSTM is trained over multiple epochs to minimize MSE and maximize the F-score, thereby balancing precision and recall.

Lastly, the number of parameters in the GRU model is significantly smaller than that of the LSTM model, making it less computationally intensive yet still effective in modeling temporal dependencies. A many-to-one GRU-based architecture is applied to weekly COVID-19 case prediction. Each GRU model processes an input vector. $x_{t}$ at time step t, similar to the LSTM, consisting of 14 entries: 13 for Google Trends search data across the 13 regions of Saudi Arabia and the remaining is for the actual number of cases in Riyadh during the same week. The GRU model utilizes reset and update gates to regulate the flow of information, efficiently maintaining information preservation while discarding irrelevant information as time advances. The GRU’s output at each time step is denoted as $h_{t}$ . The GRU model computes its hidden state using the following equations.


$$r_{t}=\sigma (W_{\mathrm{xr}}x_{t}+W_{\mathrm{hr}}h_{t-1}+b_{r})$$ (10)

$$z_{t}=\sigma (W_{\mathrm{xz}}x_{t}+W_{\mathrm{hz}}h_{t-1}+b_{z})$$ (11)

$${\tilde{h}}_{t}=\tanh(W_{\mathrm{xh}}x_{t}+W_{\mathrm{hh}}(r_{t}⊙h_{t-1})+b_{h})$$ (12)

$$h_{t}=z_{t}⊙h_{t-1}+(1-z_{t})⊙{\tilde{h}}_{t}$$ (13)

Where $x_{t}$ is the current input vector, $h_{t-1}$ is the hidden state from the previous time step, $r_{t}$ represents a reset gate, $z_{t}$ represents an update gate, ${\tilde{h}}_{t}$ is the candidate activation, $h_{t}$ is the current hidden state (output), *σ* is the sigmoid activation function, and ⊙ is the element-wise multiplication. The Equations 10–13 enable the GRU to selectively retain or discard temporal information at each step, thereby adapting to patterns in the data sequence. In the training process, several epochs are used to decrease the MSE, ensuring the model is as accurate as possible in prediction. Through the use of GRU in combination with LSTM and BiLSTM, the paper guarantees an end-to-end assessment of various RNN architectures in capturing the temporal dynamics of COVID-19 cases.

#### 2.4.2 Training period and time lag adjustment

The training period spans the whole year from January 2021 to December 2021, providing sufficient data for models to learn temporal patterns and dependencies. A critical aspect of the training process is the incorporation of time lag adjustments, which are essential when aligning model predictions with real-world scenarios. The proposed approach involves three procedural steps: Each model is initially trained on the training dataset without any temporal adjustments, allowing it to learn the patterns inherent in the data. The adjusted outputs are then classified into anomalies or non-anomalies. Following output classification, the model’s outputs are adjusted for predefined time lags, which range from 1 to 4 weeks. This adjustment involves shifting classified values forward in time to align with the actual case occurrences. Calculating the F1-score values for each shift enhances the models’ ability to predict future cases accurately. This proposed approach ensures the models are finely tuned to authentically reflect real data patterns, thereby improving both predictive reliability and effectiveness.

#### 2.4.3 Anomaly detection

Anomaly detection is the process of identifying patterns in data that deviate significantly from expected behavior (36). In this study, we focus on sequence anomalies in weekly time-series data, where sudden rises in COVID-19 case numbers reflect unexpected disruptions in disease trends (37). Early recognition of these deviations is essential for detecting potential outbreaks, making anomaly detection a critical component of the proposed framework. Among commonly used approaches—clustering, machine learning, and statistical methods—we adopt a statistical threshold-based technique due to its simplicity, interpretability, and suitability for real-time public-health monitoring where low computational overhead is required (38). In our method, anomalies are defined as weeks in which predicted cases exceed a threshold derived from the mean and standard deviation of weekly case counts. The threshold is computed using Equation 14:


Threshold=σ+kμ (14)

where μ represents the mean number of weekly cases, σ denotes the standard deviation, and k is a scaling factor controlling the strictness of anomaly detection. In this study, k was set to 1, corresponding to one standard deviation above the mean. This value was chosen based on empirical validation: preliminary sensitivity analysis showed that k = 1 provided the best balance between detecting true outbreaks and avoiding excessive false alarms. Using the predictions generated by the trained models, weekly case counts are classified as anomalous (‘1’) or normal (‘0’) depending on whether they exceed the threshold. This process leverages the temporal dependencies learned by the deep learning models, ensuring that meaningful deviations in case trends are detected accurately. By effectively marking atypical increases, the anomaly detection procedure enhances the framework’s ability to identify unexpected outbreak signals in COVID-19 case data.

## 3 Performance evaluation

The final stage of the proposed methodology involves accurately testing and evaluating the trained models to assess their performance and reliability as instruments for forecasting and anomaly detection. This process begins by using the trained models to predict the number of COVID-19 cases within the test dataset period. These predictions can then be analyzed to determine the presence of anomalies according to a previously defined threshold. Recall is defined as the ratio of correctly classified anomaly numbers [True Positives (TP)] to the total number of correctly classified and missing reported anomaly numbers [False Negatives (FN)] (39). Precision is defined as the ratio of correctly classified anomaly numbers (TP) to the total number of correctly classified and misclassified anomalies (False Positives (FP)) (40). The F1-score is defined as the harmonic mean of precision and recall (41). The metrics utilizing the Chou symbol are defined in Equations (15–17).


$$\text{Recall}=\frac{\mathrm{TP}}{\mathrm{TP}+\mathrm{FN}}$$ (15)

$$\text{Precision}=\frac{\mathrm{TP}}{\mathrm{TP}+\mathrm{FP}}$$ (16)

$$F-\text{score}=\frac{2*(\text{Recall}*\text{Precision})}{\text{Recall}+\text{Precision}}$$ (17)

MSE measures the average of the squared differences when comparing actual values and predicted values (42). A vector of n predictions is generated from a sample of n data points for all variables, and Y is the vector of observed values for the variable being predicted, with $\hat{Y}\;$ Describing the predicted values calculated in Equation 18.


$$\mathrm{MSE}=\frac{1}{n}\;\sum_{i=1}^{n}{\left(Y_{i}-\hat{Y_{i}}\right)}^{2}$$ (18)

## 4 Experimental results and analysis

In this section, we present the experimental results and discuss the findings in depth. The analysis measures the performance of three proposed neural network models (LSTM, BiLSTM, and GRU) in terms of their MSE and F1-score performance metrics. The results are discussed in terms of using Google Trends search data to forecast weekly COVID-19 case numbers and detect anomalies.

### 4.1 Models configurations

The three RNN architectures—LSTM, BiLSTM, and GRU—are trained to forecast weekly numbers of COVID-19 cases using preprocessed data. Each search term is used to train a separate model, incorporating term-specific patterns, and resulting in a total of nine differentiated models. These models are trained at a lower learning rate of 0.001, with a batch size of 32, and a maximum of 50 epochs. Early stopping is implemented to prevent overfitting, as shown in Table 2. MSE is employed as the loss function to quantify the difference between predicted and observed case counts. Time lags of from 1 to 4 weeks are introduced to study the effects of temporal shifts on prediction accuracy. A key issue in model optimization is determining the optimal value of the variable k and identifying the optimal threshold value for anomaly detection. Through empirical experimentation, we found that setting k = 1 yields the most favorable results. This value achieves an optimal compromise between sensitivity and specificity in the anomaly detection process, preventing overfitting to noise in the data while accurately detecting significant departures from the predicted case pattern.

**Table 2:** RNN model hyperparameters comparison.

Parameter	LSTM	BiLSTM	GRU
Number of layers	2	2	2
Hidden units per layer	64	64	64
Dropout rate	0.2	0.2	0.2
Learning rate	0.001	0.001	0.001
Batch size	32	32	32
Maximum epochs	50	50	50
Optimizer	Adam	Adam	Adam
Loss function	MSE	MSE	MSE
Early stopping patience	10	10	10
Time lag configurations	1–4 weeks	1–4 weeks	1–4 weeks
Total trainable parameters	~33,000	~66,000	~25,000

### 4.2 Experimental setup and anomaly threshold sensitivity

The specifications for the hardware and software necessary to allow a correct assessment and analysis are given here. The hardware environment was built using an AMD Ryzen 53,500 U processor, 8 GB DDR4 memory, 256 GB SSD storage, and an integrated Radeon Vega 8 GPU. These components guarantee efficient performance in terms of data processing and visualization. The software is executed on the Microsoft Windows 10 operating system and written in the Python programming language. There is a dominant framework for designing and training neural networks, TensorFlow, which is flexible and scalable. Scikit-learn is employed for preprocessing, evaluation, and computation of metrics, while Pandas and NumPy facilitate efficient data manipulation and numerical operations. Matplotlib supports data visualization when exploring trends and distributions. To manage the computational workload arising from processing big data across multiple regions, efficient data structures and vectorized operations are employed.

Further, to justify the selection of k=1 used for anomaly detection (introduced in Section 2.4.3), a sensitivity analysis was performed across multiple k -values: k={0.5,1,1.5,2} . For each value, anomaly labels were generated using the trained models, and detection performance was assessed against ground-truth data in terms of precision, recall, and F1-score.

Table 3 summarizes the results. It shows that k=1 provides the best balance between sensitivity and specificity. Smaller values of k (e.g., 0.5) resulted in excessive false positives, whereas larger values (e.g., 2) missed several significant anomalies. Based on this empirical evaluation, k=1 was adopted for all subsequent experiments in the manuscript.

**Table 3:** Performance of different k-values for anomaly detection.

k-value	Precision	Recall	F1-score
0.5	0.68	0.91	0.78
1	0.83	0.83	0.83
1.5	0.88	0.7	0.78
2	0.92	0.55	0.69

### 4.3 Model performance: training and testing with anomaly detection

**Figure 2:**
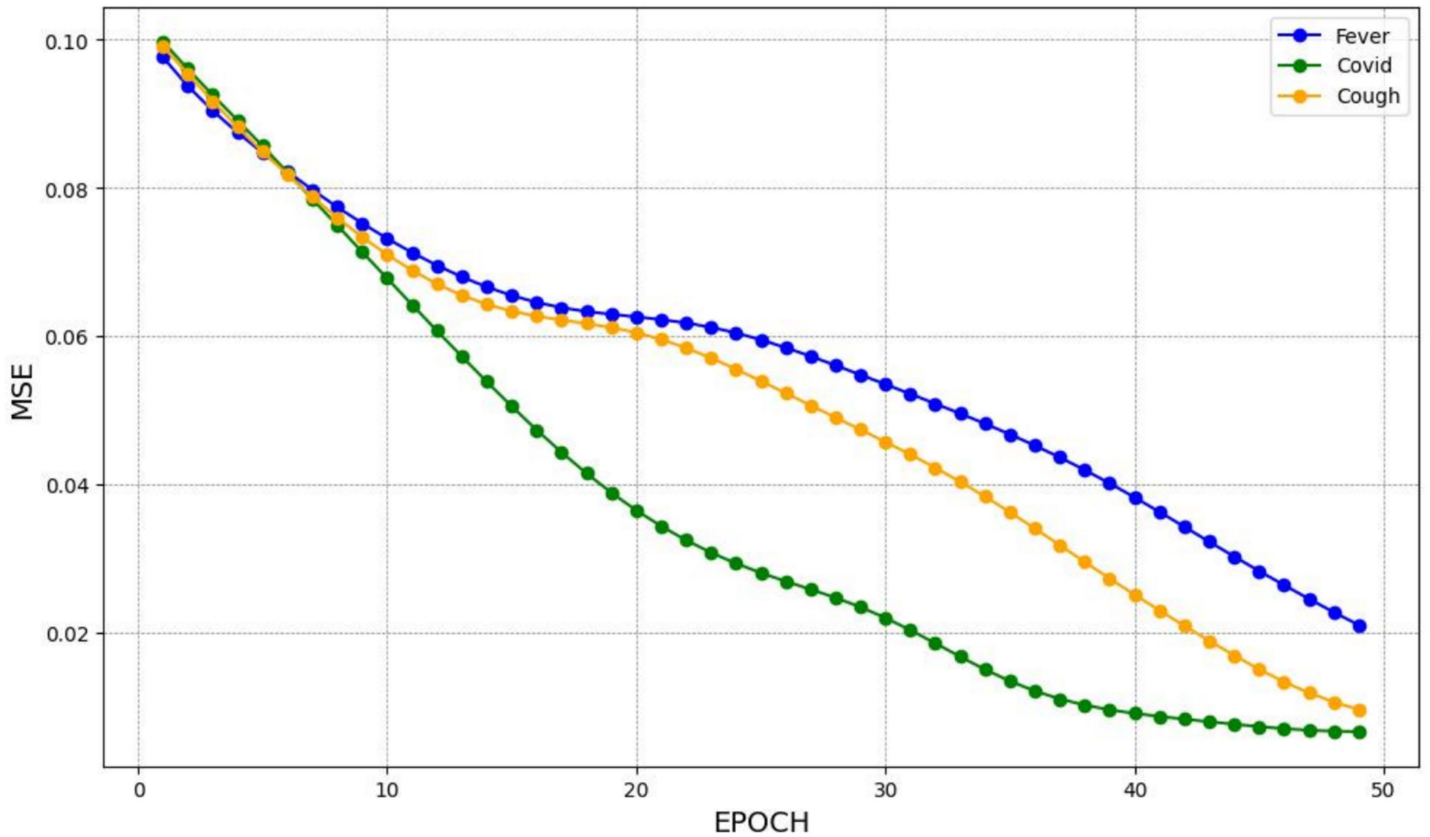
The MSE for training the LSTM model.

This subsection presents the training performance of the neural network models: LSTM, BiLSTM, and GRU. The performance of each model is evaluated based on the MSE metric over multiple epochs. The training process aims to minimize MSE, thereby optimizing the model’s predictive accuracy for weekly case numbers. The LSTM, BiLSTM, and GRU models are trained on the dataset with three primary search terms related to COVID-19: “COVID,” “cough,” and “fever.” The LSTM model exhibits a consistent reduction in MSE across all training epochs for the three search terms, as depicted in Figure 2, indicating that the model is approaching an optimal prediction. It effectively captures the temporal dynamics related to COVID-19 infections and demonstrates the potential of the approach in working with time series data that contain long-term dependencies.

**Figure 3:**
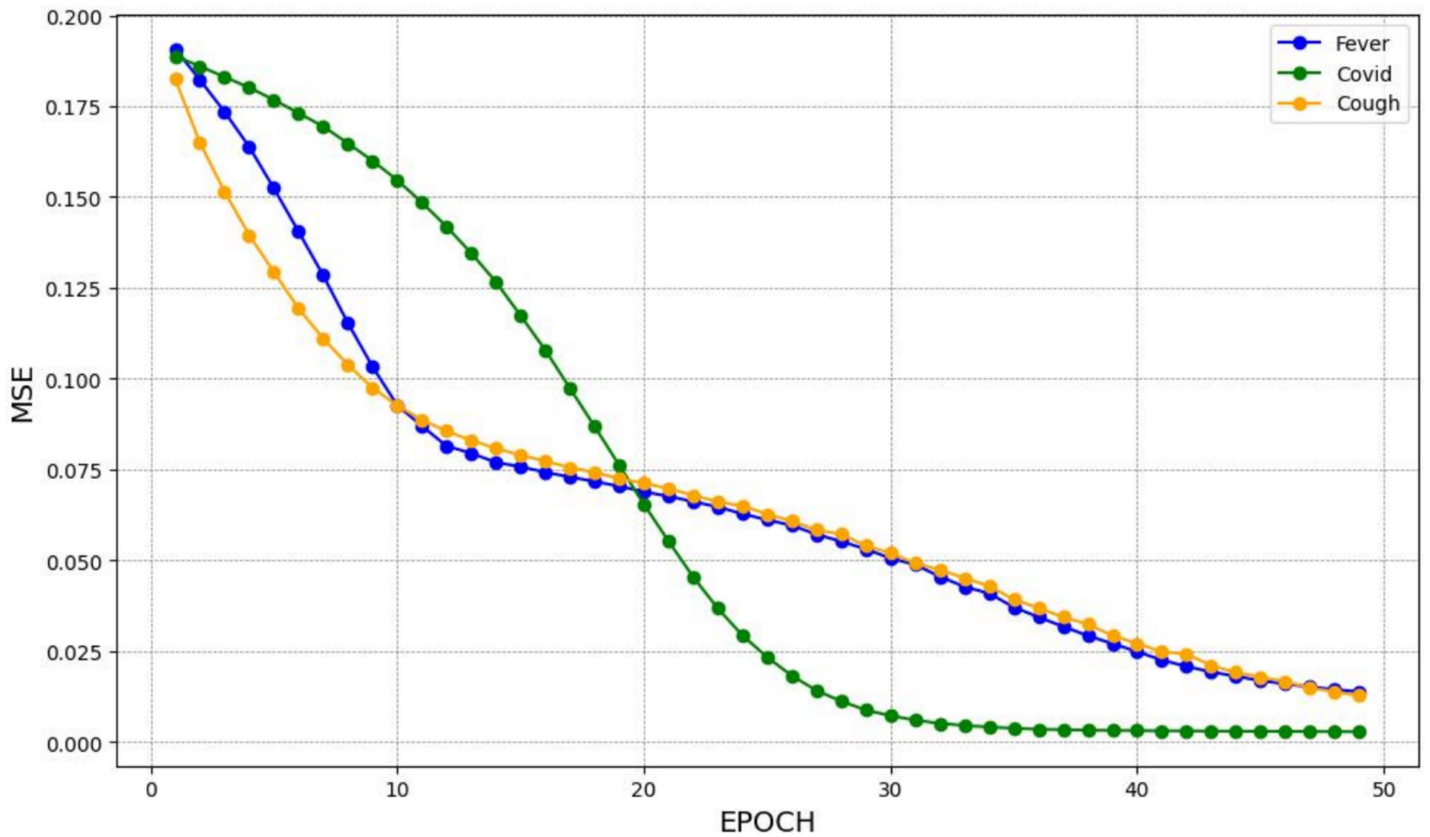
The MSE for training the BiLSTM model.

Similarly, the BiLSTM model shows decreasing MSE during the training epochs, as illustrated in Figure 3. Its bidirectional architecture enhanced its ability to learn complex patterns by capturing dependencies in both forward and backward directions, resulting in improved predictive performance.

**Figure 4:**
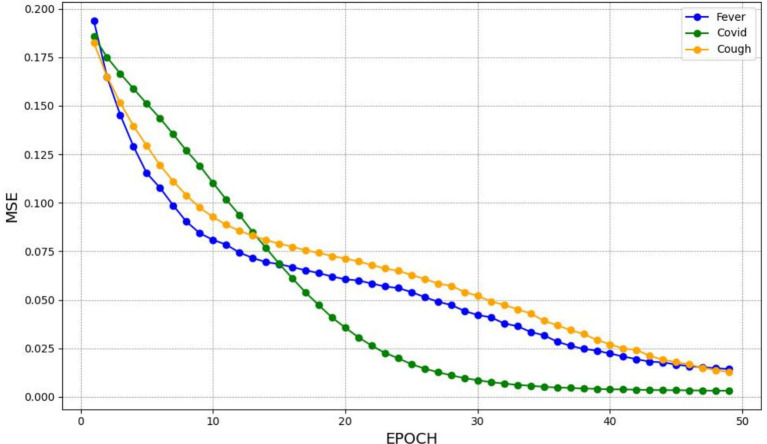
The MSE for training the GRU model.

The GRU model’s training performance, as presented in Figure 4, also demonstrates efficient learning capabilities with a steady decline in MSE, confirming its suitability for time series prediction. Its simplified architecture supports more rapid training, while maintaining accuracy. The consistent decrease in MSE across all models suggests they learn effectively from the data and improve predictive accuracy over time.

To more precisely assess the models, we also evaluated their performance in terms of anomaly detection using the F1-score over time lags ranging from 1 to 4 weeks. The F1-score balances precision and recall, giving a balanced overview of predictive performance.

**Figure 5:**
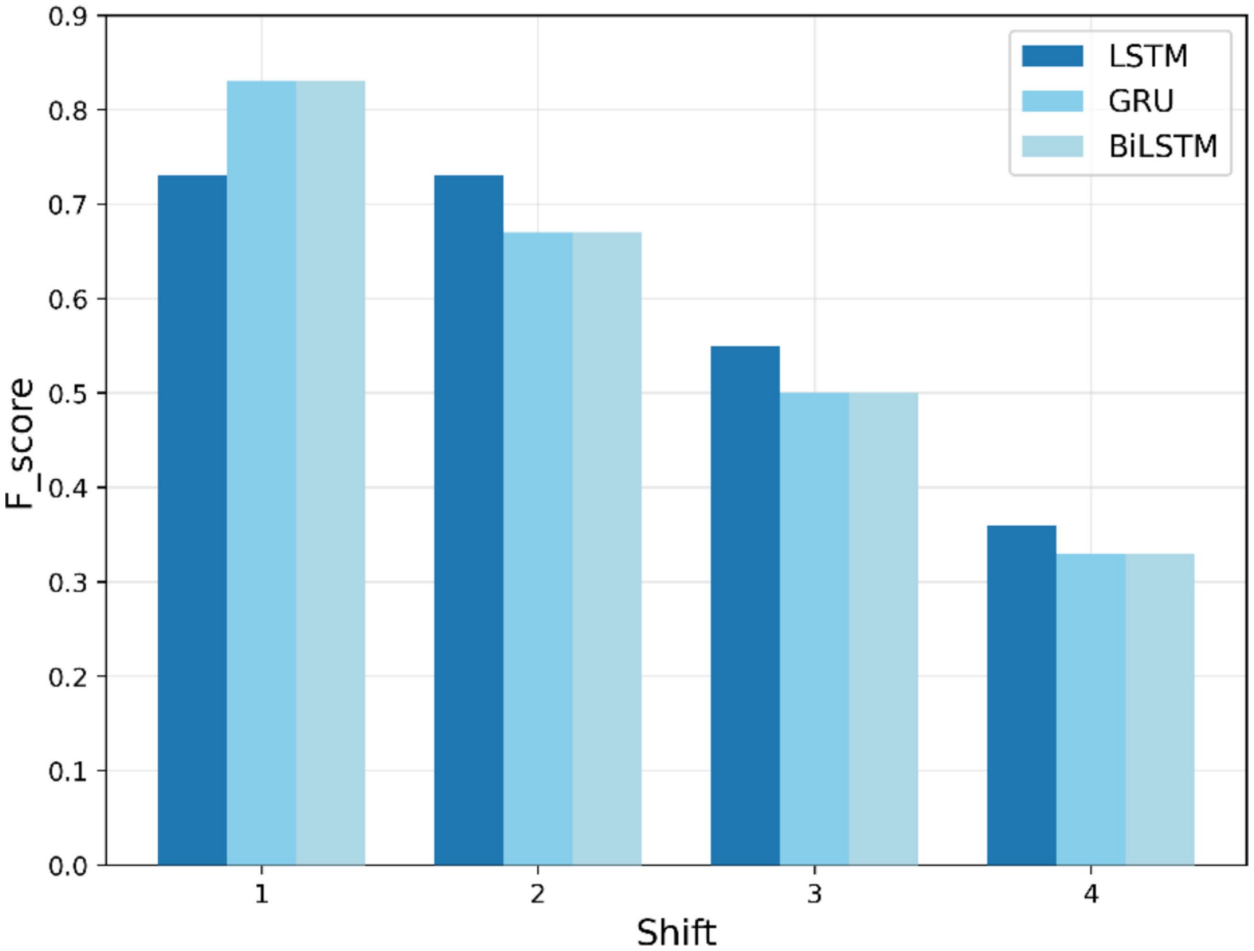
Training F1-score trends for “fever” search term.

Figure 5 describes the F1-scores obtained by the models LSTM, BiLSTM, and GRU for the “fever” search term for each shift in value during training. The F1-scores (ranging from 1 to 0.83) indicate that the GRU and BiLSTM models initially perform better than the LSTM model, with an F1-score approaching 0.83 at shift 1, and achieving slightly above 0.7 at the start. However, as the shift increases, all models exhibit a decline in performance. At shift 4, the F1-scores for the three models converge to approximately 0.33, indicating a reduction in predictive capacity as the shift values increase.

**Figure 6:**
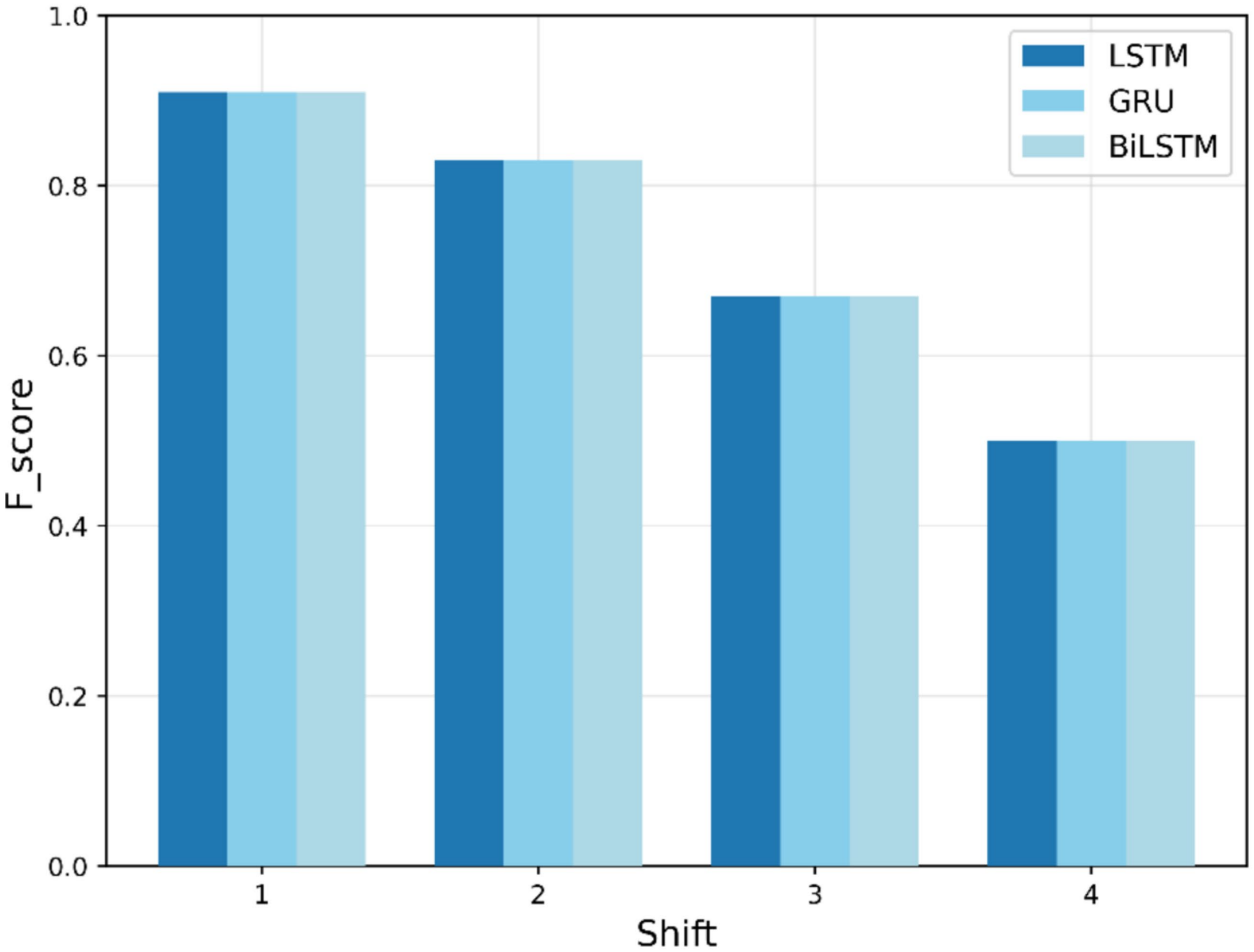
Training F1-score trends for “COVID” search term.

Figure 6 depicts the F1-scores for the LSTM, BiLSTM, and GRU models for the “COVID” search term. All models start with an F1-score of 0.91 at shift 1, indicating a perfect performance. However, as the shift increases, the F1-score gradually decreases, dropping to approximately 0.50 by shift 4. This decline highlights the model’s decreasing effectiveness when required to handle greater shifts in the data.

**Figure 7:**
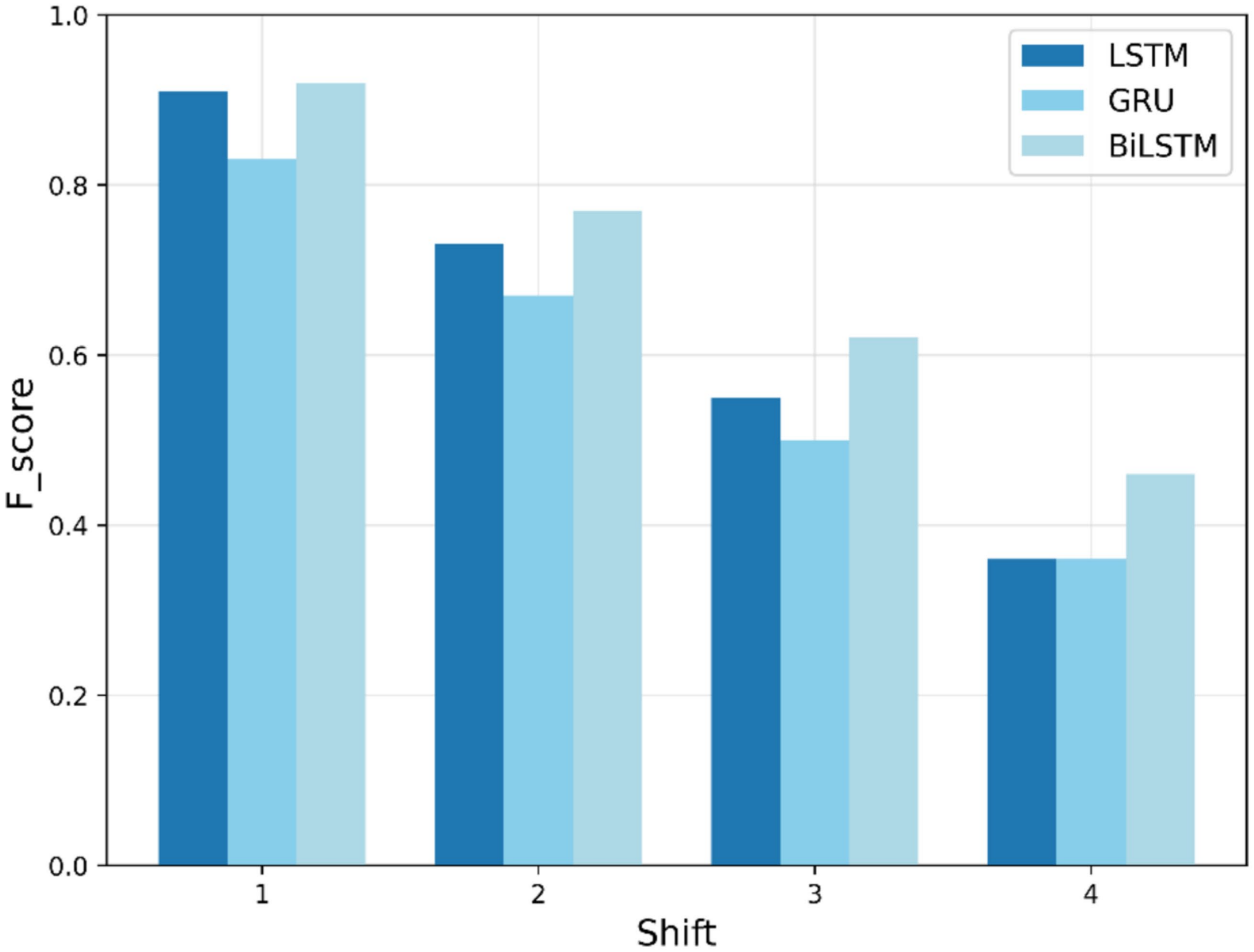
Training F1-score trends for “cough” search term.

Figure 7 depicts the F1-scores for the LSTM, BiLSTM, and GRU models’ performance when identifying the “cough” search term. Initially, all the models start with high scores between 0.92 and 0.83. As training proceeds, their scores generally decrease, suggesting they may be struggling to improve with more data. BiLSTM exhibits the slowest decline, indicating that it may be the most effective model for this task.

**Figure 8:**
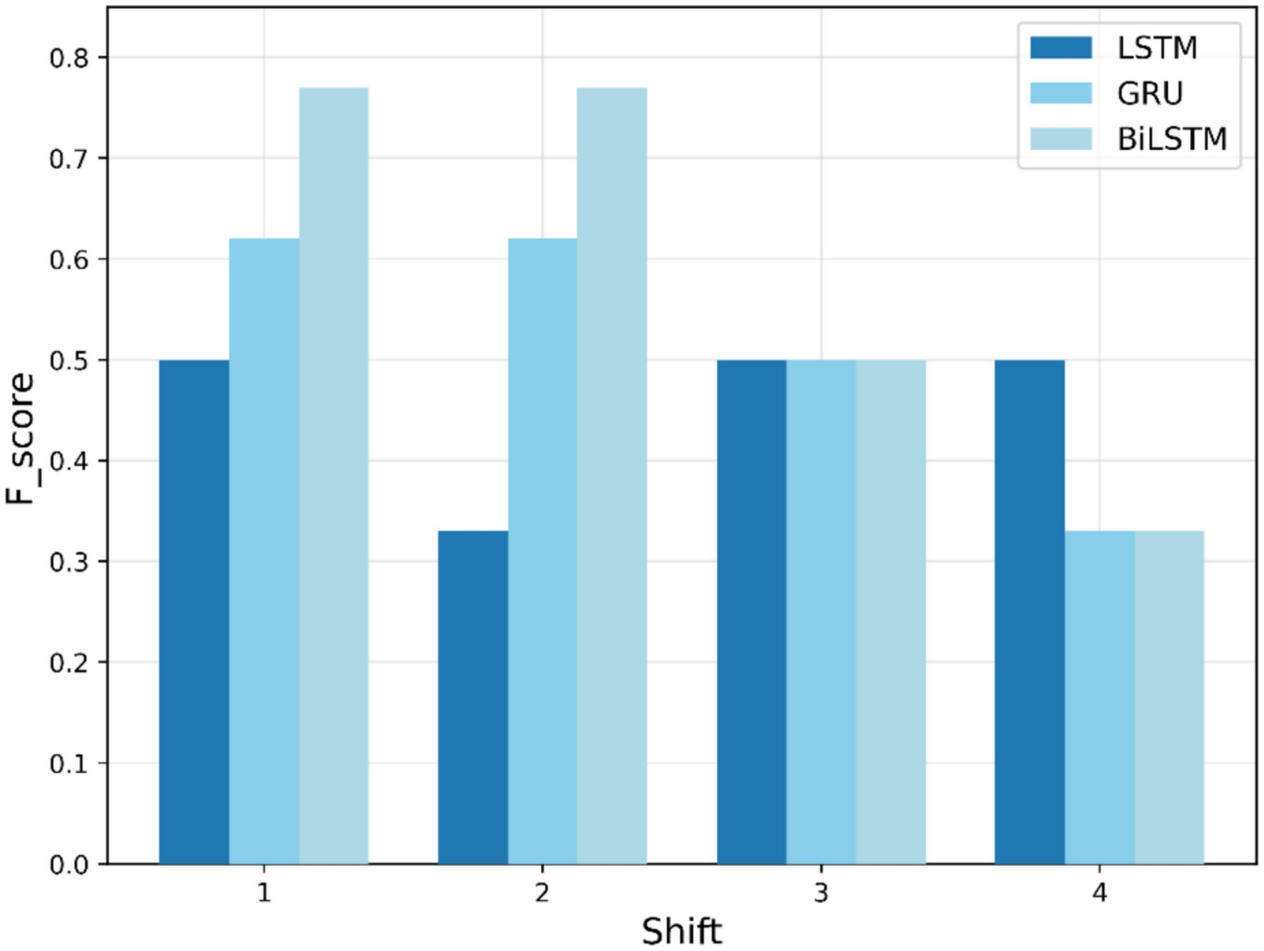
Testing F1-score trends for “fever” search term.

**Figure 9:**
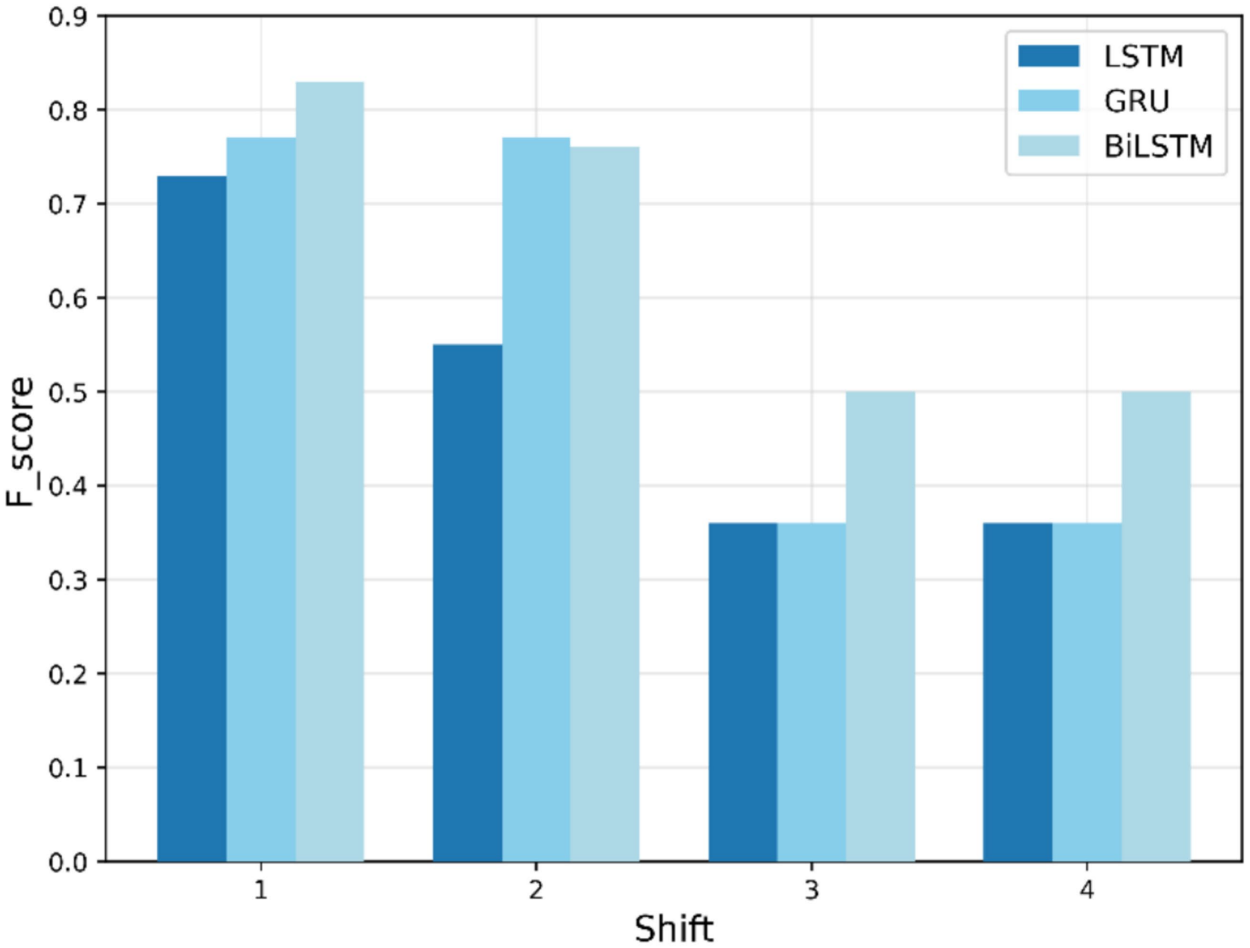
Testing F1-score trends for “COVID” search term.

**Figure 10:**
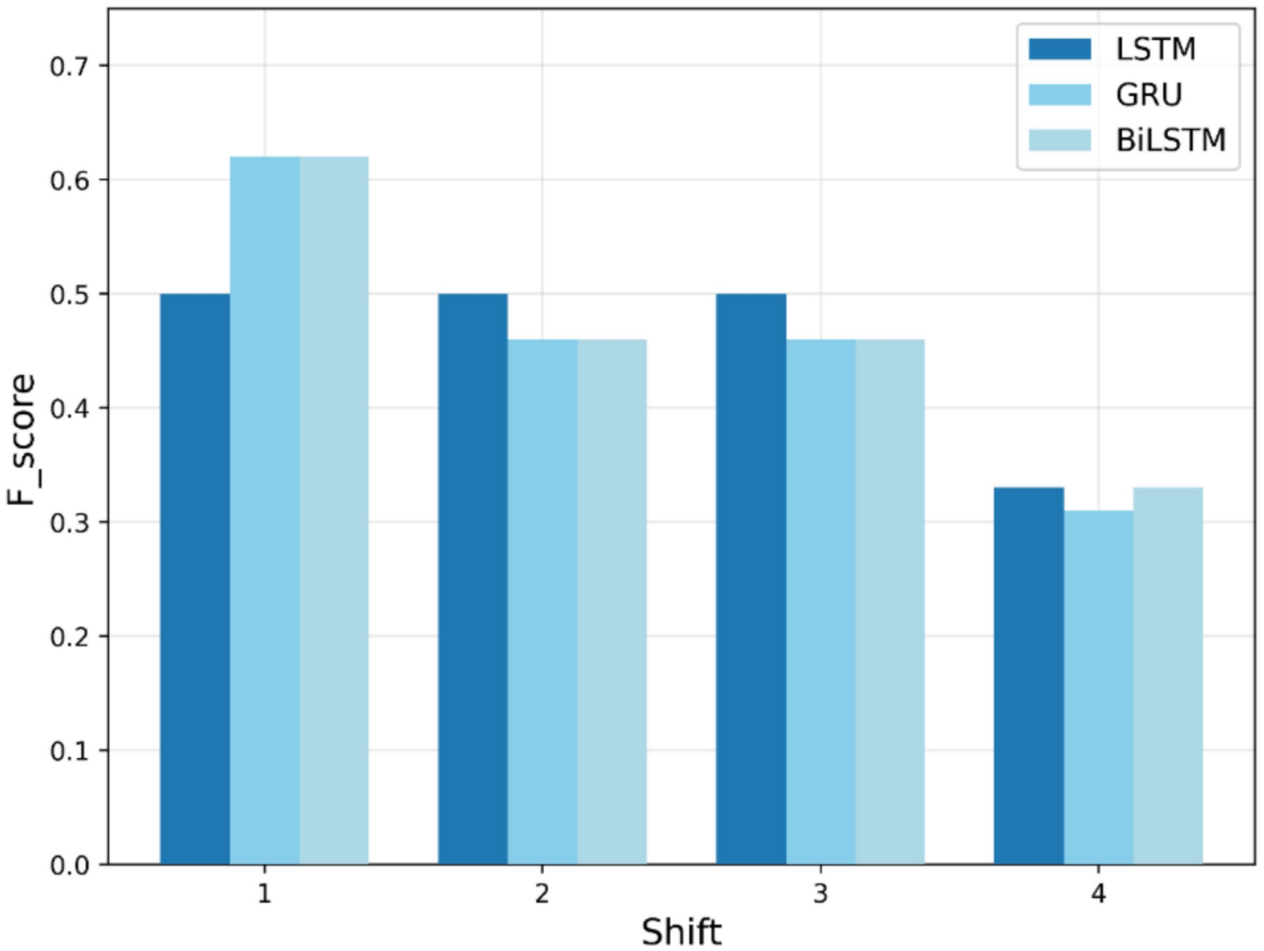
Testing F1-score trends for “cough” search term.

During the testing phase, the models are evaluated for their ability to predict COVID-19 numbers and identify anomalies in the testing dataset. Figures 8–10 show F1-scores on the testing dataset for “fever,” “COVID,” and “cough,” respectively. As depicted in Figure 8, BiLSTM shows significantly better performance in the first-phase shifts, with the highest F1-score (0.77) in both shifts 1 and 2, GRU is (0.62) behind, and the LSTM lags at (0.5) and (0.33), respectively. In shift 3, all three models yield the same results, with an F1-score of 0.5. By shift 4, performances regress, and BiLSTM and GRU scores are 0.33, while the LSTM maintains stability. In general, BiLSTM exhibits strong performance for shifts in the early periods; however, all models fail to perform well in later shifts.

As shown in Figure 9, the BiLSTM outperforms the other models, achieving the best results at shifts 1 and 2 (0.83 and 0.77, respectively). GRU then achieves a moderate performance, while the LSTM underperforms, especially in shifts 2 to 4. Each model returns lower and more equal performance in shifts 3 and 4, BiLSTM and GRU slightly worse than LSTM. The results emphasize the BiLSTM’s superiority at managing temporal variability.

According to the data given in Figure 10, GRU and BiLSTM perform better than LSTM in shift 1 (F1-score of 0.62 instead of 0.5). Performance deteriorates in shifts 2 and 3, with GRU and BiLSTM getting 0.46 and LSTM 0.5, respectively. In Shift 4, all the models reduce only slightly, with BiLSTM and LSTM at 0.33 and GRU at 0.31 being marginally lower. The findings demonstrate that GRU and BiLSTM achieve better performance compared to the previous shifts, while all models exhibit poorer performance at later stages of adaptation.

**Figure 11:**
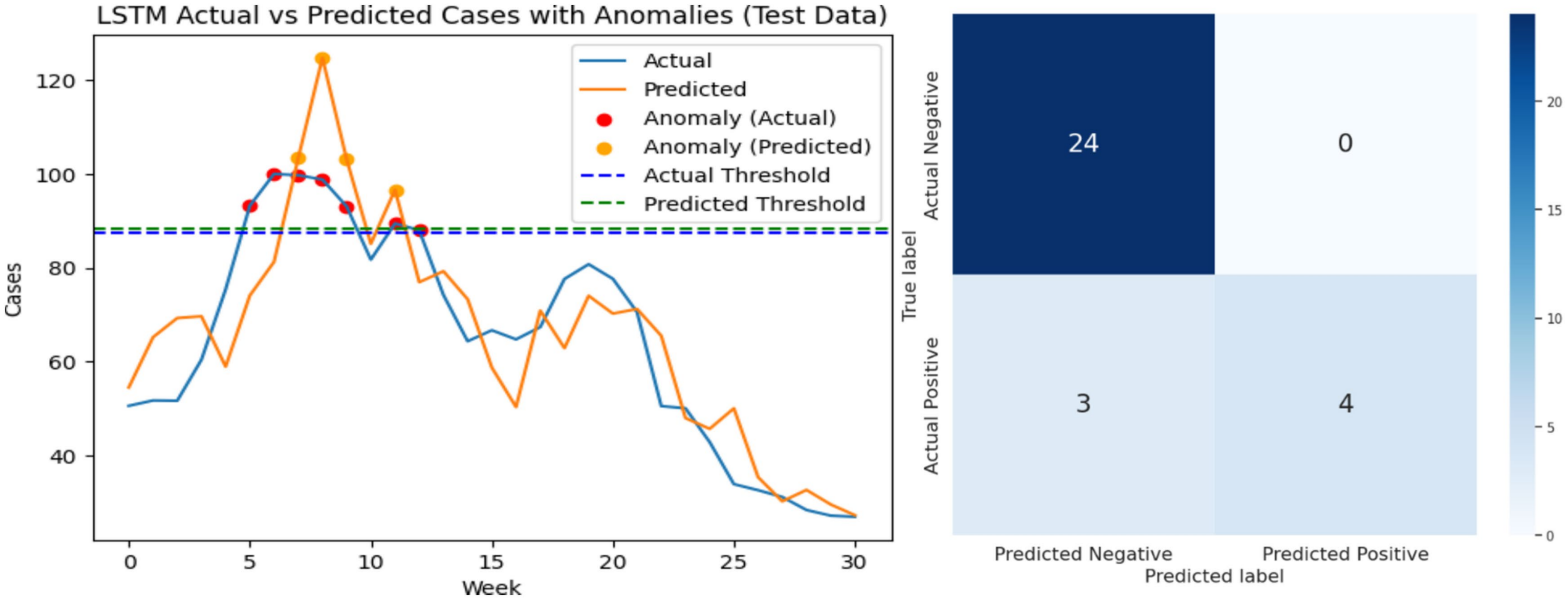
Performance analysis of the LSTM model: best result graph and confusion matrix.

**Figure 12:**
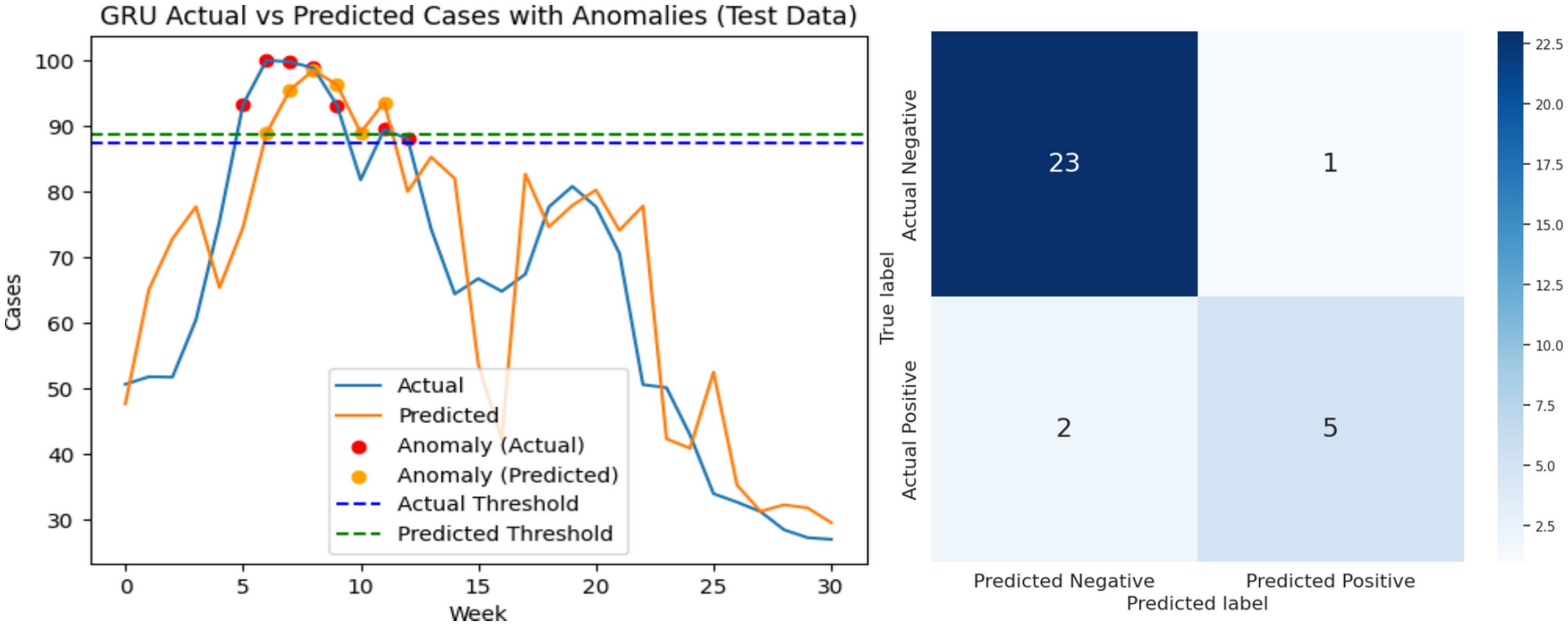
Performance analysis of the GRU model: best result graph and confusion matrix.

**Figure 13:**
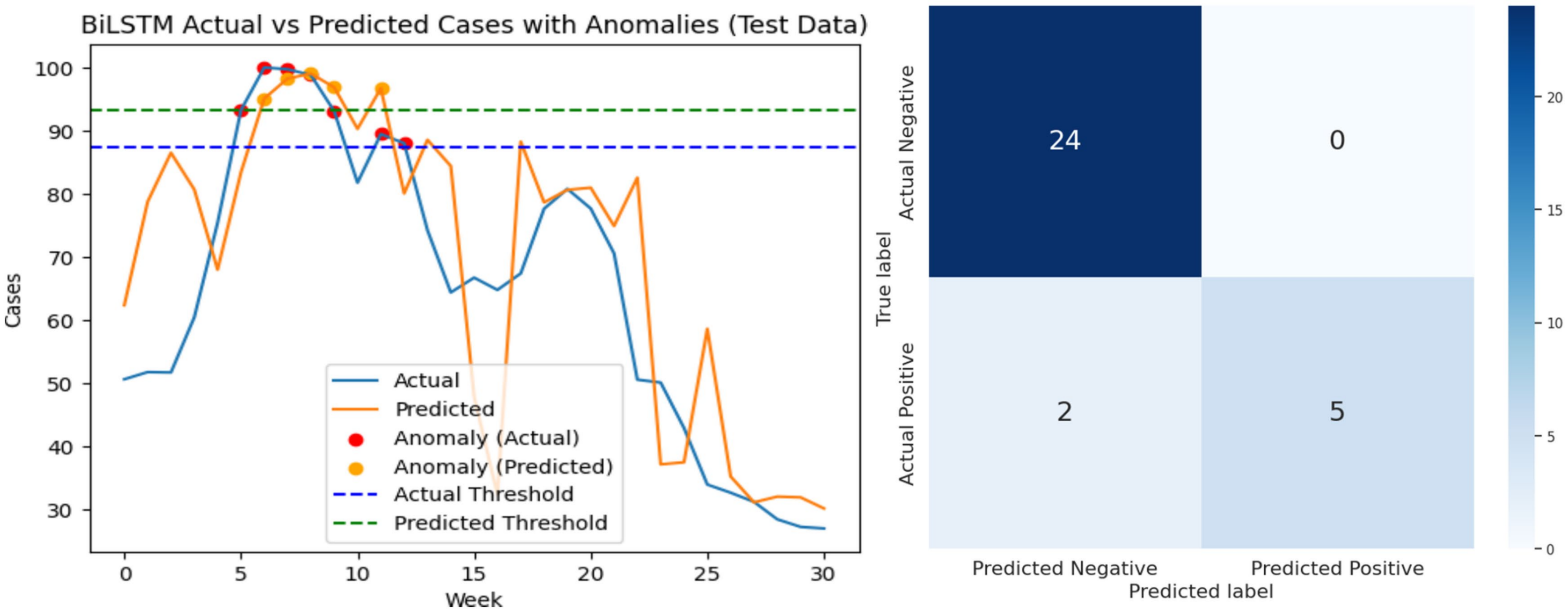
Performance analysis of BiLSTM model: best result graph and confusion matrix.

Figures 11–13 visually present the best results for the LSTM, GRU, and BiLSTM models when predicting Riyadh COVID cases at shift 1 using the search term “COVID.” Each figure includes two side-by-side graphs, showing real data and the corresponding predictions for each model, along with a confusion matrix. The confusion matrix evaluates performance metrics, categorizing predictions as true positives, true negatives, false positives, or false negatives based on thresholds specified for anomaly detection. This combined graphical and matrix-based representation provides a detailed comparison of the models’ predictive accuracy and effectiveness at identifying patterns in the data.

## 5 Discussion

The analysis of the experimental results highlights the predictive performance and efficiency of the LSTM, BiLSTM, and GRU models for forecasting COVID-19 cases and detecting anomalies based on key search term trends. The training phase demonstrates the ability of all models to minimize MSE over 50 epochs, reflecting their capacity to capture temporal patterns effectively. Of the three models, BiLSTM consistently outperforms LSTM and GRU, leveraging its bidirectional architecture to identify complex dependencies more effectively. This bidirectional processing allows the model to capture both past and future temporal relationships in weekly COVID-19 trends, detecting subtle shifts that unidirectional LSTM or the simplified GRU may miss. This capability is particularly advantageous in epidemiological forecasting, where behavioral signals in Google Trends may precede or follow actual case surges.

Table 4 summarizes the training-based F1-scores across different shifts. In training-based anomaly detection, BiLSTM exhibits better performance across various shift values, particularly for “COVID,” “fever,” and “cough” search queries, with F1-scores of 0.91, 0.83, and 0.92, respectively, at an early shift of 1 week. Nevertheless, all models show a decrease in F1-score with an elapsed time lag and converge to approximately 0.33 by the last shift. This decline is attributed to the weakening of temporal dependencies over longer forecasting horizons, making it increasingly difficult for the models to accurately predict anomalies far in advance. BiLSTM shows the slowest decrease, highlighting its robustness in the presence of temporal variability.

**Table 4:** Overall summary of training performance—F1-score comparison across time lags.

Search term	Model	Shift 1	Shift 2	Shift 3	Shift 4
Fever	LSTM	0.73	0.73	0.55	0.36
	BiLSTM	0.83	0.67	0.50	0.33
	GRU	0.83	0.67	0.50	0.33
COVID	LSTM	0.91	0.83	0.67	0.50
	BiLSTM	0.91	0.83	0.67	0.50
	GRU	0.91	0.83	0.67	0.50
Cough	LSTM	0.91	0.73	0.55	0.36
	BiLSTM	0.92	0.77	0.62	0.46
	GRU	0.83	0.67	0.50	0.36

Moreover, Table 5 presents testing-based F1-scores across time lags. The testing phase further validates the models’ capabilities, with BiLSTM maintaining a consistent advantage, particularly for the “COVID” search term, for which it achieves an F1-score of 0.83 at an early shift. GRU performs competitively at early shifts, demonstrating adaptability in anomaly detection tasks, while LSTM slightly lags in accuracy but maintains stability across shifts.

**Table 5:** Overall summary of testing performance—F1-score comparison across time lags.

Search term	Model	Shift 1	Shift 2	Shift 3	Shift 4
Fever	LSTM	0.50	0.33	0.50	0.33
	BiLSTM	0.77	0.77	0.50	0.33
	GRU	0.62	0.62	0.50	0.33
COVID	LSTM	0.73	0.55	0.36	0.36
	BiLSTM	0.83	0.76	0.50	0.50
	GRU	0.77	0.77	0.36	0.36
Cough	LSTM	0.50	0.50	0.50	0.33
	BiLSTM	0.62	0.46	0.46	0.33
	GRU	0.62	0.46	0.46	0.31

Analysis of false positives and false negatives provides additional practical insight. A false positive corresponds to predicting an outbreak when actual cases remain normal, which could trigger unnecessary public health alerts. Conversely, a false negative corresponds to missing an actual spike, potentially delaying intervention measures. BiLSTM’s superior F1-scores at early shifts indicate fewer critical errors, making it more reliable for early-warning systems. Figures 11–13 visually present the best results for LSTM, GRU, and BiLSTM at shift 1 using the “COVID” search term. Confusion matrices and predictive graphs confirm that BiLSTM strikes a balance between predictive accuracy and robust anomaly detection, particularly in periods where temporal dependencies are strongest.

In summary, BiLSTM demonstrates enhanced predictive performance due to its bidirectional structure, whereas all models face challenges in higher time-lag predictions. Understanding false positives and negatives underscores the practical relevance of model predictions for real-world epidemiological monitoring.

## 6 Conclusion

This study systematically evaluates the performance of LSTM, BiLSTM, and GRU models for predicting COVID-19 case numbers and detecting anomalies using time-series data derived from Google search trends. All three models effectively capture temporal patterns, demonstrating the potential of integrating behavioral data with epidemiological records for epidemic monitoring. Among them, BiLSTM consistently outperforms LSTM and GRU by leveraging bidirectional temporal dependencies, which allows it to more accurately model complex fluctuations in case numbers. In contrast, LSTM and GRU offer faster training times, making them advantageous for real-time applications where computational efficiency is critical. The decline in predictive performance with increasing time lags highlights the inherent challenge of modeling long-term dependencies in highly dynamic datasets, underscoring the need for robust strategies to maintain accuracy over extended horizons. Despite its higher computational cost, BiLSTM’s superior predictive power positions it as a valuable tool for early outbreak detection and anomaly identification.

Future work will focus on improving scalability and efficiency through hybrid model architectures and attention mechanisms, while integrating additional contextual data sources such as population mobility, vaccination rates, and policy interventions. Deploying these enhanced models in real-world healthcare systems will enable real-time monitoring, early warning, and data-driven decision-making, ultimately supporting more proactive and effective epidemic management (43).

## Data Availability

The original contributions presented in the study are included in the article/supplementary material, further inquiries can be directed to the corresponding author.

## References

[1] Lampos V Miller AC Crossan S Stefansen C. Advances in nowcasting influenza-like illness rates using search query logs. Sci Rep. (2015) 5:12760. doi: 10.1038/srep12760, 26234783 PMC4522652

[2] Yang S Santillana M Kou SC. Accurate estimation of influenza epidemics using Google search data via ARGO. Proc Natl Acad Sci. (2015) 112:14473–8. doi: 10.1073/pnas.1515373112, 26553980 PMC4664296

[3] Prasanth S Singh U Kumar A Tikkiwal VA Chong PH. Forecasting spread of COVID-19 using google trends: a hybrid GWO-deep learning approach. Chaos, Solitons Fractals. (2021) 142:110336. doi: 10.1016/j.chaos.2020.110336, 33110297 PMC7580652

[4] Jimenez AJ Estevez-Reboredo RM Santed MA Ramos V. COVID-19 symptom-related Google searches and local COVID-19 incidence in Spain: correlational study. J Med Internet Res. (2020) 22:e23518. doi: 10.2196/23518, 33156803 PMC7757783

[5] Strzelecki A Azevedo A Albuquerque A. Correlation between the spread of COVID-19 and the interest in personal protective measures in Poland and Portugal. Health. (2020) 8:203. doi: 10.3390/healthcare8030203, 32659922 PMC7551869

[6] Walker MD Sulyok M. Online behavioural patterns for coronavirus disease 2019 (COVID-19) in the United Kingdom. Epidemiol Infect. (2020) 148:e110. doi: 10.1017/S0950268820001193, 32498731 PMC7306408

[7] Lampos V Majumder MS Yom-Tov E Edelstein M Moura S Hamada Y . Tracking COVID-19 using online search. NPJ Digital Med. (2021) 4:17. doi: 10.1038/s41746-021-00384-w, 33558607 PMC7870878

[8] Niu B Liang R Zhang S Zhang H Qu X Su Q . Epidemic analysis of COVID-19 in Italy based on spatiotemporal geographic information and Google trends. Transbound Emerg Dis. (2021) 68:2384–400. doi: 10.1111/tbed.13902, 33128853

[9] Schuster B Tizek L Schielein MC Ziehfreund S Rothe K Spinner CD . Retracing the COVID-19 pandemic in Germany from a public perspective using Google search queries related to "coronavirus". Das Gesundheitswesen. (2021) 83:e9–e14. doi: 10.1055/a-1398-5417, 33862647

[10] Morris M Hayes P Cox IJ Lampos V. Neural network models for influenza forecasting with associated uncertainty using web search activity trends. PLoS Comput Biol. (2023) 19:e1011392. doi: 10.1371/journal.pcbi.1011392, 37639427 PMC10491400

[11] Ahmad I Flanagan R Staller K. Increased internet search interest for GI symptoms may predict COVID-19 cases in US hotspots. Clin Gastroenterol Hepatol. (2020) 18:2833–4. doi: 10.1016/j.cgh.2020.06.058 32629121 PMC7834024

[12] Rajan A Sharaf R Brown RS Sharaiha RZ Lebwohl B Mahadev S. Association of search query interest in gastrointestinal symptoms with COVID-19 diagnosis in the United States: infodemiology study. JMIR Public Health Surveill. (2020) 6:e19354. doi: 10.2196/19354, 32640418 PMC7371406

[13] Yuan X Xu J Hussain S Wang H Gao N Zhang L. Trends and prediction in daily new cases and deaths of COVID-19 in the United States: an internet search-interest based model. Exploratory Res Hypothesis Med. (2020) 1:1–6. doi: 10.14218/ERHM.2020.00023, 32348380 PMC7176069

[14] Abbas M Morland TB Hall ES El-Manzalawy Y. Associations between google search trends for symptoms and COVID-19 confirmed and death cases in the United States. Int J Environ Res Public Health. (2021) 18:4560. doi: 10.3390/ijerph18094560, 33923094 PMC8123439

[15] Husnayain A Chuang T-W Fuad A Su EC-Y. High variability in model performance of Google relative search volumes in spatially clustered COVID-19 areas of the USA. Int J Infect Dis. (2021) 109:269–78. doi: 10.1016/j.ijid.2021.07.031, 34273513 PMC8922685

[16] Yousefinaghani S Dara R Mubareka S Sharif S. Prediction of COVID-19 waves using social media and Google search: a case study of the US and Canada. Front Public Health. (2021) 9:656635. doi: 10.3389/fpubh.2021.656635, 33937179 PMC8085269

[17] Effenberger M Kronbichler A Shin JI Mayer G Tilg H Perco P. Association of the COVID-19 pandemic with internet search volumes: a Google TrendsTM analysis. Int J Infect Dis. (2020) 95:192–7. doi: 10.1016/j.ijid.2020.04.033, 32305520 PMC7162745

[18] Ayyoubzadeh SM Ayyoubzadeh SM Zahedi H Ahmadi M Kalhori SRN. Predicting COVID-19 incidence through analysis of google trends data in Iran: data mining and deep learning pilot study. JMIR Public Health Surveill. (2020) 6:e18828. doi: 10.2196/18828, 32234709 PMC7159058

[19] Satpathy P Kumar S Prasad P. Suitability of Google trends™ for digital surveillance during ongoing COVID-19 epidemic: a case study from India. Disaster Med Public Health Prep. (2023) 17:e28. doi: 10.1017/dmp.2021.249, 34343467 PMC8460424

[20] Venkatesh U Gandhi PA. Prediction of COVID-19 outbreaks using google trends in India: a retrospective analysis. Healthcare Info Res. (2020) 26:175–84. doi: 10.4258/hir.2020.26.3.175, 32819035 PMC7438693

[21] Xie T Tan T Li J. An extensive search trends-based analysis of public attention on social media in the early outbreak of COVID-19 in China. Risk Manag Healthcare Policy. (2020) 13:1353–64. doi: 10.2147/RMHP.S257473, 32943953 PMC7468945

[22] Li C Chen LJ Chen X Zhang M Pang CP Chen H. Retrospective analysis of the possibility of predicting the COVID-19 outbreak from internet searches and social media data, China, 2020. Eurosurveillance. (2020) 25:2000199. doi: 10.2807/1560-7917.ES.2020.25.10.2000199, 32183935 PMC7078825

[23] Schnoell J Besser G Jank BJ Bartosik TJ Parzefall T Riss D . The association between COVID-19 cases and deaths and web-based public inquiries. Infect Dis Ther. (2021) 53:176–83. doi: 10.1080/23744235.2020.1856406, 33287607

[24] Su K Xu L Li G Ruan X Li X Deng P . Forecasting influenza activity using self-adaptive AI model and multi-source data in Chongqing, China. EBioMedicine. (2019) 47:284–92. doi: 10.1016/j.ebiom.2019.08.024, 31477561 PMC6796527

[25] Murayama T. Wakamiya S. Aramaki E., "Single Model for Influenza Forecasting of Multiple Countries by Multi-task Learning", *Joint European Conference on Machine Learning and Knowledge Discovery in Databases*, (2021), pp. 335–350.

[26] Boulesnane A Meshoul S Aouissi K. Influenza-like illness detection from Arabic Facebook posts based on sentiment analysis and 1D convolutional neural network. Mathematics. (2022) 10:4089. doi: 10.3390/math10214089

[27] Yom-Tov E Lampos V Inns T Cox IJ Edelstein M. Providing early indication of regional anomalies in COVID-19 case counts in England using search engine queries. Sci Rep. (2022) 12:2373. doi: 10.1038/s41598-022-06340-2, 35149764 PMC8837788

[28] Dimick JB Ryan AM. Methods for evaluating changes in health care policy: the difference-in-differences approach. JAMA. (2014) 312:2401–2. doi: 10.1001/jama.2014.16153, 25490331

[29] Wagner M Lampos V Yom-Tov E Pebody R Cox IJ. Estimating the population impact of a new pediatric influenza vaccination program in England using social media content. J Med Internet Res. (2017) 19:e416. doi: 10.2196/jmir.8184, 29269339 PMC6257312

[30] City Population, (2024), "Saudi Arabia: Regions & Major Cities - Population Statistics, Maps, Charts, Weather and Web Information", Available online at: https://www.citypopulation.de/en/saudiarabia/cities/ (Accessed on February 5, 2024).

[31] Sherstinsky A. Fundamentals of recurrent neural network (RNN) and long short-term memory (LSTM) network. Physica D Nonlinear Phenom. (2020) 404:132306. doi: 10.1016/j.physd.2019.132306

[32] Aldhyani TH Alkahtani H. A bidirectional long short-term memory model algorithm for predicting COVID-19 in gulf countries. Life. (2021) 11:1118. doi: 10.3390/life11111118, 34832994 PMC8625101

[33] ArunKumar K Kalaga DV Kumar CMS Kawaji M Brenza TM. Forecasting of COVID-19 using deep layer recurrent neural networks (RNNs) with gated recurrent units (GRUs) and long short-term memory (LSTM) cells. Chaos, Solitons Fractals. (2021) 146:110861. doi: 10.1016/j.chaos.2021.110861, 33746373 PMC7955925

[34] Google Trends. (2023). Available online at: https://trends.google.com/trends/explore (Accessed on 24-Oct-2023).

[35] Saudi Arabia Coronavirus disease (COVID-19) situation, King Abdullah Petroleum Studies and Research Center (KAPSARC) Riyadh, Minstry of Health. (2024).

[36] Chandola V Banerjee A Kumar V. Anomaly detection: a survey. ACM Comput Surv. (2009) 41:1–58. doi: 10.1145/1541880.1541882

[37] Schmidl S Wenig P Papenbrock T. Anomaly detection in time series: a comprehensive evaluation. Proc VLDB Endow. (2022) 15:1779–97. doi: 10.14778/3538598.3538602

[38] Braei M Wagner S. Anomaly detection in univariate time-series: a survey on the state-of-the-art. arXiv. (2020) doi: 10.48550/arXiv.2004.00433

[39] Uddin I Al Qahtani SA Noor S. Deep-m6Am: a deep learning model for identifying N6, 2′-O-Dimethyladenosine (m6Am) sites using hybrid features. AIMS Bioeng. (2025) 12:145–61. doi: 10.3934/bioeng.2025006

[40] Noor S AlQahtani SA Khan S. Chronic liver disease detection using ranking and projection-based feature optimization with deep learning. AIMS Bioeng. (2025) 12:50–68. doi: 10.3934/bioeng.2025003

[41] Almusallam N Khan S Alarfaj FK Ahmad N. A robust deep learning framework for RNA 5-methyluridine modification prediction using integrated features. BMC Biol. (2025) 23:328. doi: 10.1186/s12915-025-02433-2, 41174762 PMC12577295

[42] Khan S Dilshad N Ahmad N Noor S AlQahtani SA. Integrating AI in security information and event management for real time cyber defense. Sci Rep. (2025) 15:35872. doi: 10.1038/s41598-025-19689-x, 41087388 PMC12521385

[43] Alturki R Munshi A Alshawi B Agarwal K Khan F Khan S. CardioBERT: a cardiac identification using fusion features in consumer healthcare. IEEE Trans Consum Electron. (2025) 71:3522–30. doi: 10.1109/TCE.2025.3575522

